# Mucinous Carcinoma, Mucinous Borderline Tumor and Pseudomyxoma Ovarii in a Cystic Teratoma: A Histological Conundrum

**DOI:** 10.3390/diagnostics15151957

**Published:** 2025-08-04

**Authors:** Cinzia Giacometti, Mariateresa Mirandola, Camillo Aliberti, Filippo Molinari, Lisa Marcolini, Daniele Mautone, Guido Martignoni

**Affiliations:** 1 Pathology Unit, Pederzoli Hospital, Peschiera del Garda, 37019 Verona, Italy; lisa.marcolini@ospedalepederzoli.it (L.M.); guido.martignoni@univr.it (G.M.); 2 Gynecology & Obstetrics Unit, Pederzoli Hospital, Peschiera del Garda, 37019 Verona, Italy; mariateresa.mirandola@outlook.com (M.M.); daniele.mautone@ospedalepederzoli.it (D.M.); 3 Radiology Unit, Pederzoli Hospital, Peschiera del Garda, 37019 Verona, Italy; camillo.aliberti@ospedalepederzoli.it; 4 Academic Unit of Obstetrics and Gynecology, IRCSS Hospital Polyclinic San Martino, 16132 Genoa, Italy; filippo.molinari2@gmail.com; 5 Pathology Unit, Department of Diagnostic and Public Health, University of Verona, 37126 Verona, Italy

**Keywords:** mature cystic teratoma, mucinous lesions, mucinous carcinoma

## Abstract

Mature teratomas account for approximately 20% of all ovarian tumors identified in pathological studies. Benign or malignant somatic neoplasms developing within teratomas can arise from any tissue in up to 2% of mature cystic teratomas, including low-grade malignant mucinous neoplasms. This report presents the case of a 34-year-old woman with no previous gynecological or general health issues, who was admitted to our Hospital after an asymptomatic pelvic mass was detected during a routine exam. A transvaginal ultrasound revealed a unilateral pelvic mass in the left adnexal region, measuring 8 cm. The CT scan showed a cystic-appearing formation measuring nearly 12 cm, which indented the bladder dome. Final diagnosis indicated a mucinous carcinoma arising from a mucinous borderline lesion within the context of a mature ovarian teratoma. No other involvement or lymphadenopathies were detected on 18FDG-PET CT scan, and the patient is now well and free of recurrences.

**Figure 1:**
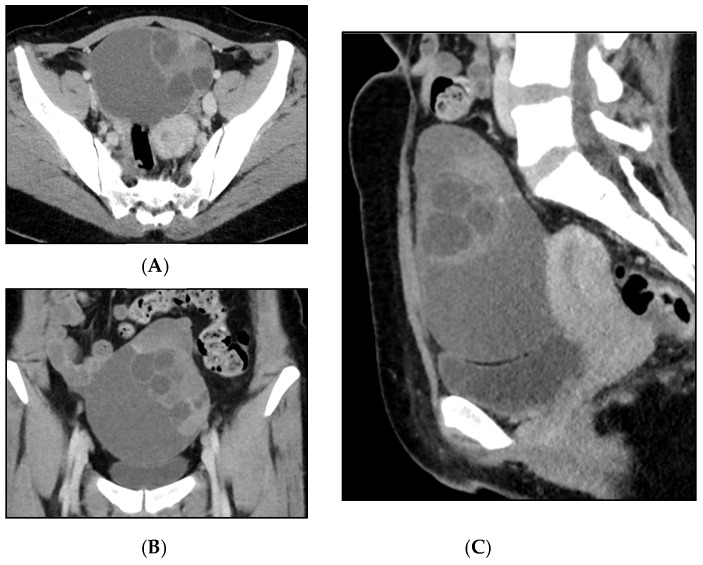
Mature teratomas represent approximately 20% of all ovarian tumors identified in pathological studies. Somatic neoplasms that develop within teratomas are either benign or malignant, arising from any tissue present in the teratomas. These somatic neoplasms may be found in up to 2% of mature cystic teratomas, although they are extremely rare in immature teratomas [1,2]. Epithelial neoplasms can occur in up to 2% of dermoid cysts, with squamous cell carcinoma accounting for the majority (approximately 80%) of malignant tumors. They exhibit a high mutational burden, with mutations in the TP53 gene being the most common abnormality [3,4,5]. Adenocarcinoma is the second most common carcinoma in teratomas (7%), while sarcoma accounts for 8%. Melanomas, melanocytic nevi, and basal cell carcinomas are much less common [6]. In the literature, other benign and malignant tumors (usually as single case reports) have also been reported, including sebaceous carcinoma [7], urothelial carcinoma [7], and low-grade malignant mucinous neoplasms [8]. Mucinous ovarian neoplasms associated with teratomas (MONATs) are uncommon neoplasms, with only a few large series published to date [9,10,11]. Histologically, MONATs are a heterogeneous group of neoplasms, conventionally classified as cystoadenoma, borderline mucinous tumor, and mucinous carcinoma [9,10]. The origin of MONATs has been proposed to be either independent of the teratoma (i.e., having a Mullerian-type phenotype) or derived from the germ cell components of the tumor (with a typical intestinal/appendiceal-type phenotype) [11]. MONATs may be associated with pseudomyxoma ovarii and peritonei, although these characteristics are more frequently observed in a metastatic context [8,12,13]. We present the case of a 34-year-old multiparous woman with no previous gynecological or general health issues. She had been regularly undergoing annual gynecological exams and Pap smear tests. In May 2024, she was admitted to our Hospital after an asymptomatic pelvic mass was detected during a gynecological routine examination. During transvaginal ultrasound performed by an expert operator, a unilateral pelvic mass in the left adnexal region was diagnosed. The lesion was a multilocular solid mass (fewer than 10 locules), measuring 80 mm in maximum diameter, with irregular internal margins due to a solid component measuring 14 × 25 mm, containing mixed content, and moderately vascularized at color Doppler (color score 3). No normal ovarian parenchyma was observed; the right ovary and uterus appeared normal, and no signs of ascites or free fluid in Douglas’s pouch were detected. Serum oncological markers—including CA 125, CA 19.9, CEA, CA 15.3, and AFP—were all negative. IOTA adnex model showed an 84.2% risk of benign tumor, a 15.8% risk of malignancy, and a 7.6% risk of a borderline tumor. A surgical path was decided for the patient, a laparoscopic visualization with frozen section of the left adnexa was planned due to the risk of malignancy and borderline tumor diagnosis driven by transvaginal ultrasound examination; the patient was counseled for eventual surgical staging or debulking surgical procedures based on frozen section diagnosis, according to the most recent guidelines [14,15]. A total body computed tomography with a contrast medium was performed before surgery to evaluate the chest and abdomen. The CT scan was performed with a 64-slice Siemens Somatom Definition AS with dedicated software for dose reduction (SAFIRE, software versione *syngo* CT 2011A (VA40). The exam was performed before and after the administration of the contrast medium. The contrast medium (90 cc 1.5 pro/kg) was Iomeron 400. (**A**) Coronal and (**B**) axial viewing of a cystic-appearing formation (measuring 109 × 77 × 123 mm) was reported in the left adnexal region. The cyst appeared as multiloculated with internal septa and an endoluminal solid projection (characterized by post-contrastographic enhancement). (**C**) On sagittal viewing, the ovarian mass was responsible for the imprint on the bladder dome, without wall infiltration. There were no expansile formations in the right adnexal site. Anteverted-flexed uterus was of regular volume. No free effusion in the abdomen and no other signs of distant metastasis were reported.

**Figure 2:**
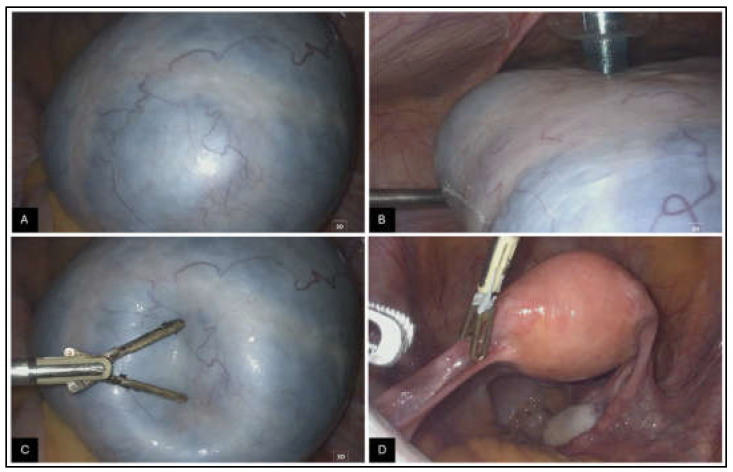
The surgical laparoscopy confirmed the presence of a mass in the left adnexa. The video of this laparoscopic procedure is in the Supplementary Material. The right adnexa, uterus, appendix, pelvic and abdominal peritoneum, and upper abdominal organs appeared normal. No bulky lymph nodes were detected. Intraoperative images of the ovarian mass: The external surface of the lesion appeared smooth, well-defined, and whitish (**A**,**B**), with reduced consistency and a cystic content (**C**). The maximum diameter was approximately 12 cm. The contralateral ovary and the uterus appeared normal (**D**).

**Figure 3:**
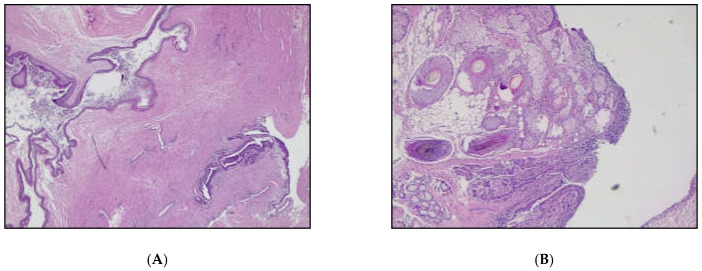
A left adnexectomy was performed without spillage through endobag extraction and sent for frozen section analysis. The frozen section identified both cystic and solid nodules. The diagnosis of a borderline mucinous tumor within the context of a mature ovarian teratoma was made. Surgical staging was completed based on frozen section diagnosis with an appendectomy, multiple biopsies of the peritoneum and omentum, endometrial biopsies, and peritoneal washing. The final diagnosis revealed foci of low-grade mucinous carcinoma (well-differentiated–G1), associated with a mucinous borderline tumor (mBOT) and intraovarian mucin accumulation (pseudomyxoma ovarii) arising in a mature ovarian teratoma. Pseudomixoma ovarii is characterized by pools of mucin dissecting the ovarian stroma. Less commonly, in the context of ovarian mucinous tumors, it can be associated with pseudomyxoma peritonei. In the latter case, it can be challenging to distinguish from low-grade appendiceal carcinoma. Assessing the status of the appendix is mandatory in MONATs. Pseudomyxoma peritonei is characterized by the presence of mucin deposits and/or mucinous epithelium in the peritoneum, and it is usually associated with mucin-producing neoplasms, typically originating from the appendix (in 80% of cases) [16,17]. (**A**) Dermoid cyst (teratoma) component (H&E, original magnification 25×); (**B**) Mature teratoma component, showing pilosebaceous unis glands, and adipose tissue (H&E, original magnification 50×).

**Figure 4:**
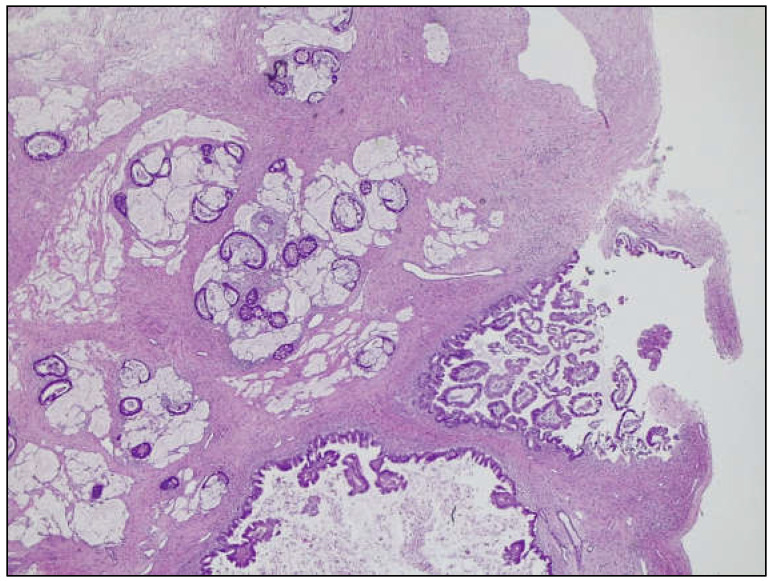
The cyst harbored an MBOT and a low-grade mucinous carcinoma, admixed with an extracellular mucin pool, i.e., pseudomyxoma ovarii (H&E, original magnification 25×). The carcinomatous component confidently represented the solid projection seen in the US and CT exams, as it constituted the nodular component sampled during the frozen section exam.

**Figure 5:**
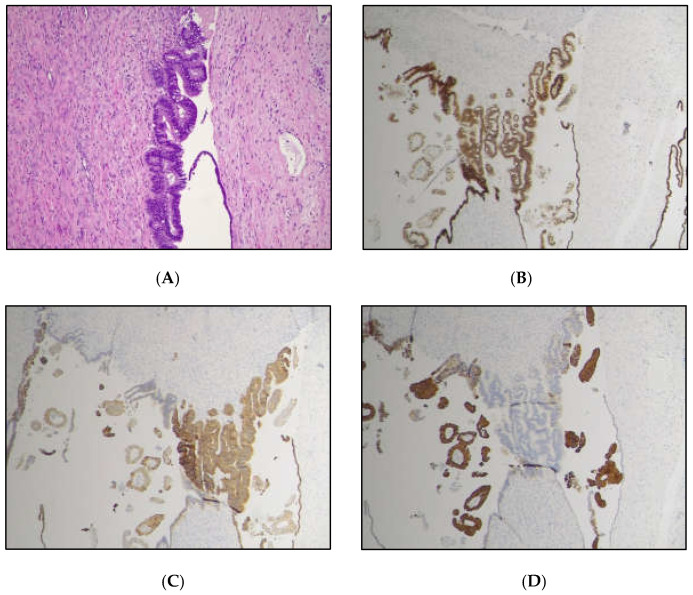
The diagnostic workup was completed with immunohistochemistry, which showed CK7 and CDX2 immunoreactivity, with negative CK20 in the mBOT, and a CK20 focal positivity along with negative CK7 and SATB2 immunolabeling in the carcinomatous component, indicating a Müllerian/surface epithelium origin, as described in the literature [1]. Neuroendocrine markers (Chromogranin A and Synaptophysin) to rule out ovarian mucinous carcinoid [18] were negative. Ovarian mucinous borderline component (Original magnification 100×); (**A**) Hematoxylin and Eosin; (**B**) CDX2 positive; (**C**) CK7 positive; (**D**) CK20 negative.

**Figure 6:**
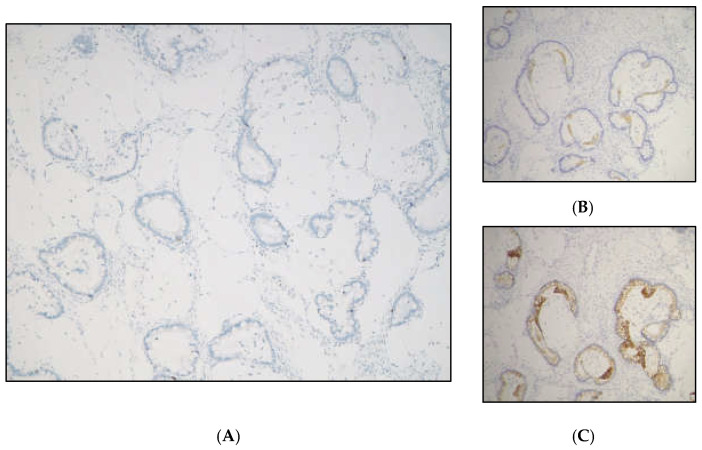
The mucinous carcinoma immunophenotype was as follows: (**A**) SATB2 negative, (**B**) CK7 negative, and (**C**) CK20 focally positive (Original magnification 100×). The remaining material was negative for peritoneal implant or localization. In the Table below (Table S1), we summarized the findings to provide an immunohistochemical workup if a MONAT is found in the ovary [16]. Due to the final definitive diagnosis of mucinous carcinoma, completion of surgical staging was planned with laparoscopic right adnexectomy and hysterectomy; lymphadenectomy could be considered because of the infiltrative pattern. After extensive counseling of the patient, due to the low-grade nature of the tumor itself, we decided to perform an 18F FDG PET CT scan to specifically exclude lymph nodes or other distant metastasis and eventually omit surgical lymphadenectomy. The 18FDG-PET CT scan was negative. Gastroscopy and colonoscopy were also performed to exclude other distant primary tumor sites, which yielded negative findings. A laparoscopy hysterectomy with right adnexectomy was then performed. Histological definitive diagnosis of the stadiation surgery specimen was free of tumor localization. The case was discussed during the Gynecological Oncological Multidisciplinary Tumor Board, and the patient was then sent for follow-up. A 3-month CT scan was negative for recurrence, and oncological serum markers were negative. The gynecological examination and transvaginal ultrasound were negative. Hormonal replacement therapy was prescribed, and a subsequent 3 to 4-month follow-up check was planned according to the most recent guidelines [14]. Neoplasms arising in mature cystic teratoma may present with abdominal swelling or acute abdominal pain or can be an incidental finding, as in the presented case. Histological classification may be highly challenging. We present a case where the histological differential diagnosis of mucinous lesions of the ovary was especially difficult, as it includes the entire spectrum of primary mucinous lesions (mucinous cystadenoma, mBOT, mucinous carcinoma); intestinal-type adenocarcinoma originating from intestinal-type epithelium within the teratoma; mucinous carcinoid arising from neuroendocrine cells; and metastatic mucinous neoplasia (colorectal carcinoma, mucinous lesions from the appendix) [18]. Mucinous lesions of Müllerian or surface epithelial origin typically show CK7 positivity and variable CK20 expression (ranging from negative to positive and from focal to diffuse), but are consistently negative for SATB2 [1,13]. SATB2 positivity can help identify the gastroenteric phenotype, and the differential diagnosis should account for intestinal-type adenocarcinoma arising from intestinal-type teratomatous epithelium or metastasis from the lower gastrointestinal tract. The diagnostic evaluation of these diverse yet histologically similar lesions must always consider the possibility of a metastatic origin, along with the clinical context. However, clinical features may not always be present, as we described in the current case report. The surgical decision to complete the assessment of the origin of mucinous neoplasms cannot be made in the intraoperative setting, as we have illustrated; the overlap of different lesions originating from various sites is very high. In the present case, the clinical and histological features concurred in defining the final diagnosis. It is pivotal to remember that patients with teratoma-associated mucinous tumors are reported to be younger than those with non-teratoma-associated mucinous neoplasms and other ovarian cancer histotypes, such as serous, endometrioid, and clear cell carcinomas [1]. In the present case, preoperative imaging led to the hypothesis of borderline/malignant nature of the lesion, highlighting the need for an expert ultrasonographer and a specialized radiologist. Teratomas are frequent findings in young patients, and malignant lesions arising in this context are rare and usually microscopic. Not all ovarian teratomas need to be surgically treated to eradicate or diagnose a potential malignant lesion. Patients at risk may be adequately identified through high-quality imaging, such as expert transvaginal ultrasound and a thorough clinical examination. No specific ultrasound signs have yet been proposed for the identification of teratoma-associated tumors; however, dimension, vascularization, and the presence of a solid component may lead to a diagnostic hypothesis. Initial surgical evaluation and a two-step surgery plan may be helpful in management: immunohystochemistry, specific diagnosis among the previouslt described wide spectrum of mucinous teratoma-associated and non-associated ovarian tumors, and their specific growth pattern can be reached only after definitive pathological evaluation and not on frozen section. Since patients affected by teratomas and teratoma-associated tumors are often young and of childbearing age, indications for surgical staging and treatment may change after final histology, such as for fertility sparing treatment if possible. Follow-up must subscribe to mucinous tumor follow-up guidelines [19]. Moreover, we showed how much preoperative diagnosis of ovarian masses may be highly challenging, and most patients are diagnosed only after pathological and immunohistochemical evaluations and a thorough differential diagnostic workup. Early detection and timely management remain crucial to improving patient outcomes. With the increasing number of reported cases, radiologists, pathologists, and clinicians must be aware of the potential for malignant transformation in typical teratomatous contexts.

## Supplementary Materials

The following supporting information can be downloaded at: https://zenodo.org/records/15801336. accessed on 27 July 2025. Video description: This supplementary video shows a laparoscopic procedure performed for the evaluation of an ovarian mass. The surgical steps include left salpingo-oophorectomy, appendectomy, peritoneal biopsies, and omental biopsy. The procedure is part of the diagnostic workup of a suspected ovarian neoplasm. https://zenodo.org/records/16737108. accessed on 27 July 2025. Table S1, describes the immunohistochemical findings in ovarian mucinous neoplasia.
